# The Potential of Disabled Infectious Single Cycle (DISC) Virus Platforms for Next Generation African Swine Fever Vaccine Development

**DOI:** 10.1155/tbed/8573171

**Published:** 2025-07-14

**Authors:** Fan Jia, Stacey E. Lynch, David T. Williams

**Affiliations:** CSIRO, Australian Centre for Disease Preparedness, 5 Portarlington Road, East Geelong, Victoria, Australia

**Keywords:** African swine fever, disabled infectious single cycle, essential ASFV genes, vaccine development and challenges

## Abstract

African swine fever (ASF) is an emergency animal disease causing significant socio-economic impacts in affected areas on a global scale. While the first generation of ASF live-attenuated virus (LAV) vaccines to be recently approved for use in some countries offer potential to kerb the spread of ASF, non-live next-generation vaccines offer a safer alternative that can also be administered to animals in ASF-free zones. Among the next-generation vaccine platforms, disabled infectious single cycle (DISC) viruses are a promising replication-incompetent viral vaccine approach. In this review, we evaluate potential ASF virus gene targets that have been shown to have essential roles in the replication cycle and could be selected as deletion targets for producing DISC vaccines. We also summarise ASF virus genes for which there is evidence for a role in replication but have not yet been examined for their essential functions. Anticipated challenges for the development of an ASF DISC vaccine include limited cell substrates for development and manufacturing, genomic and phenotypic diversity of ASFV and potential for recombination events with co-infecting field viruses leading to reversion to virulence.

## 1. Introduction

African swine fever (ASF) is a devastating disease of pigs, associated with a high mortality rate of nearly 100% for highly virulent strains. ASF has had a profound impact on the populations of domestic and wild pig species, thereby representing a threat to pig production, food security and biodiversity [1]. ASF is caused by the ASF viruses (ASFV), the single viral species belonging to the genus *Asfivirus*, family *Asfarviridae*. Since it was first identified in East Africa in the early 1900s, ASFV have evolved, historically at least 24 genotypes have been recognised based on the ASFV major capsid protein p72 gene, although it has been proposed to reduce these to six genotypes [2–4]. Genotype 2 ASFV is responsible for the current global pandemic [5–8]. ASFV has been one of the most challenging pathogens to develop vaccines against due to its complexity and tropism for immune cells. It is an enveloped, double-stranded DNA virus with a genome length varying between 170 and 190 kb. Encoded within this genome are between 151 and 167 open reading frames that are responsible for the synthesis of proteins integral to the virus's ability to attach to host cells, facilitate replication and productive infection and evade the host's immune response [9]. Based on the severity of clinical signs developed in domestic pigs after infection, ASFVs can be grouped into high, moderate and low virulence strains [10].

Decades of research and development have been conducted on ASF vaccines since the first inactivated ASFV was developed in the 1960s [11]. Other conventional approaches for vaccine development, such as attenuation by cell culture adaptation and recombinant protein-based vaccines have also been investigated for ASF; however, all candidates were found to have unsatisfactory safety profiles and insufficient immunogenicity (e.g., [12–15]). Similarly, novel approaches using DNA vaccines, alone or in combination with recombinant ASFV proteins or live attenuated virus (LAV), have failed to induce protective immunity [16–19]. Viral vectored vaccine approaches have shown more promise, with pooled antigens delivered by different viral vectors for prime and booster vaccinations protecting pigs from a lethal dose of genotype 1 ASFV [20]. However, a similar approach taken for genotype 2 ASFV was not protective [21], highlighting the need for a better understanding of the protective immune responses to ASFV to develop safe and effective subunit vaccines.

While the immunological correlates of protection for ASFV are not well understood, studies have shown that both neutralising antibodies and T cells are crucial for achieving full protection, indicating that induction of both humoral and cell-mediated immunity are important properties of an effective ASF vaccine [22, 23]. Research on the immunogenicity of different ASFV isolates has led to the identification of a range of antigenic viral proteins and epitopes that elicit specific immune responses [24]. An in silico approach also predicted immunogenic B and T cell epitopes from ASFV antigens that may have a role in eliciting protective immunity [25]. These proteins and the genes that encode them have the potential to be incorporated into delivery vectors for the development of vaccines.

## 2. First Generation Genetically Modified Live Attenuated Vaccines

With the advancement of molecular technologies for gene cloning and editing, attenuation of ASFV has been achieved by targeted gene(s) knockout [26, 27]. Viral genes selected for deletion have generally been those related to virulence and host immune system evasion. Examples of this type of attenuated vaccine are listed in Table 1. They have been developed using prototype or well-characterised virulent laboratory isolates belonging to either genotypes 1 or 2 and immunisation of pigs with these recombinant viruses has been effective to protect against lethal challenge with parental strains [28–34]. One of these modified LAVs, ASFV-G-ΔI177L, developed by targeted deletion of a late transcribed viral gene *I177L*, has been recently approved by the Vietnamese Ministry of Agriculture and Rural Development with the trade name NAVET-ASFVAC [28, 35]. Another LAV, ASFV-G-ΔMGF, developed by removing six genes from multigene family (MGF) 360 or 505 [30], and manufactured by AVAC has also been approved for use in Vietnam, as well as by the Philippines government for controlled use [36, 37]. To achieve large-scale commercial production without using primary cells, the LAV ASFV-G-ΔI177L/ΔLVR has been developed by adaptation of ASFV-G-ΔI177L in the continuous Plum Island porcine epithelial cell line (PIPEC), which resulted in a further genome deletion of 10,842 bp in the left variable region (LVR) [38]. This LAV derivative, manufactured by DABACO Group has been licenced by the Vietnamese Department of Animal Health and Production with the trade name DACOVAC-ASF2 as the third commercially available ASF vaccine [36]. The implementation of LAVs represents substantial progress for the control of ASF. However, there are safety concerns around the implementation of these first generation LAVs, such as side effects in immunised pigs causing ASF clinical symptoms, persistent infection after vaccination, horizontal transmission to cohabitated naive pigs, reversion to virulence and impact on reproductive performance of sows [28–30, 32, 33, 39], as well as the potential for recombination with co-circulating wild type strains.

Furthermore, LAVs are not an appropriate biosecurity measure for regions and jurisdictions that are free from ASF due to the risks of reversion to virulence and threat to ASF-free status [40]. Non-live or replication defective next-generation vaccines may be considered for application in such scenarios, e.g., to protect pig farming or susceptible wild suid populations in countries bordering an ASF-affected or endemic country.

## 3. Disabled Infectious Single Cycle (DISC) Virus as a Safe and Effective Vaccine for Viral Pathogens

The DISC virus approach offers an alternative candidate platform for the development of next-generation ASF vaccines. A DISC virus is a defective virus that executes only one cycle of replication in infected host cells, producing non-infectious virus particles due to the lack of an essential viral gene(s) [41]. This single cycle of viral replication prevents further spread within the host, while allowing expression of the array of viral proteins required for the generation of robust immunity.

The development of a DISC virus by genetic engineering typically involves the deletion of one or more genes coding for key proteins that are associated with viral replication. A complementary cell line expressing the missing viral gene(s) is developed in parallel as a substrate to rescue the replication of the DISC virus and for manufacturing purposes. DISC viruses have been developed as vaccines or vaccine delivery platforms from several pathogenic viruses.

There has only been a single reported attempt for the generation of a DISC ASFV [42]. In this study, a Vero cell-adapted strain (BA71V) was used as a parental virus with deletion of the viral *A104R* gene, encoding a histone-like protein (pA104R) that is essential to ASFV replication and incorporating a *GUS* gene for X-Gluc selection [42, 43]. Complementary Vero and COS-1 cells stably expressing the deleted viral protein were constructed to recover the DISC virus. However, propagation of a DISC ASFV using this approach could not be achieved beyond three passages, with wild type virus outcompeting the recombinant deletion mutants [42]. Nevertheless, this study demonstrated the feasibility of a DISC approach for ASFV vaccine development.

A DISC vaccine against herpes simplex virus type 2 (HSV-2), “Dl5-29”, was developed by deleting two viral genes *UL5* and *UL29*, which encode DNA helicase and single-stranded DNA-binding protein, respectively [41]. The rescue cell line (V529) for complementing the growth of Dl5-29 was constructed by inserting HSV-2 *UL5* and HSV-1 *UL29* into the Vero cell genome. Mutation or deletion of one or both of the viral genes resulted in a defective HSV-2 DISC vaccine candidate that provided effective protection against a lethal challenge with wild type HSV-2 in mouse models [44]. Sanofi Pasteur has manufactured Dl5-29 as a herpes vaccine candidate and confirmed its safety and ability to produce neutralising antibodies or immune cells in vaccinees in a phase I clinical trial [45].

The Sementis Copenhagen Vector (SCV) is one of the latest advancements for vaccinia virus (VACV) as a smallpox vaccine and may also be used as a general vaccine delivery platform technology. Instead of targeting viral DNA replication at the early phase of infection, SCV has been developed by deleting a gene encoding the scaffold protein D13 required for the assembly of virions to stop the production of infectious progeny [46]. The production of SCV is uniquely achieved in its rescue cell line, which is derived from Chinese hamster ovary (CHO) cells, a well-established cell substrate for manufacturing [47]. Host range restriction of SCV in CHO cells is resolved by expression of the cowpox host range gene *CP77*, while viral assembly, maturation and egress are enabled by expression of *D13L* [46]. Preclinical studies for the SCV vaccines have confirmed its safety in immunocompromised mice and efficacy in generating protective immunity against infections with mousepox, a mouse version of lethal smallpox or other pathogens of interest when SCV was applied as a vaccine vector (e.g., for chikungunya virus, Zika virus and severe acute respiratory syndrome coronavirus 2) [46, 48, 49].

The production of DISC viruses has also been applied to combat infectious diseases in livestock. Bluetongue virus (BTV) DISC vaccines have been developed by disabling an enzymatic protein, VP6, that is associated with viral RNA replication and packaging [50]. The genetic engineering approach used for generating this DISC BTV involved the introduction of several stop codons into the target gene to stop expression, improving the genetic stability of the DISC virus mutants [51, 52]. The helper cell line to rescue the amplification of DISC BTVs is BHK-21 cells expressing the VP6 protein [50]. These DISC BTV vaccines showed promising performance in animal studies, eliciting long-lasting protective immunity in both cattle and sheep against wild type virus challenge [51–53].

By removal of only essential genes to stop viral replication, immune responses induced by DISC viruses are expected to be comparable to replicating viruses. The potential for robust vaccine induced immunity coupled with the safety profile are key features that make the DISC vaccine platform attractive for next-generation ASF vaccine development.

## 4. Development of a DISC Vaccine for ASF

An important element of developing a DISC virus is to identify the key viral gene(s) that are essential to the replication cycle. Other important considerations include levels of viral antigens that are produced from a single cycle of replication and whether this will be sufficient to induce long-lasting protective immunity against subsequent infection with wild type virus, as well as safety for vaccinated animals. Advancements in understanding the ASFV replication cycle have provided a basis for selecting target genes for deletion.

**Figure 1:**
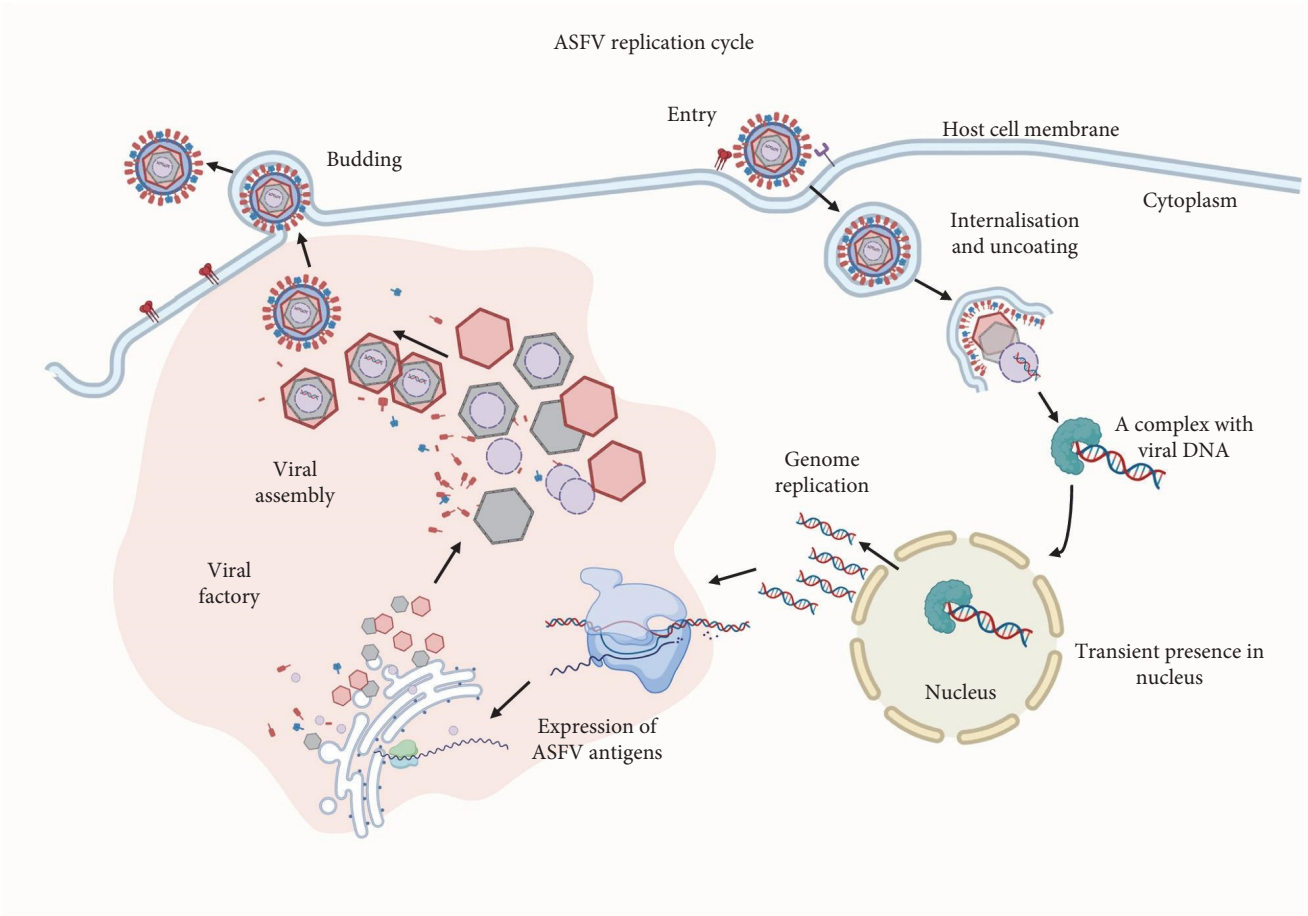
Replication cycle of African swine fever virus. The ASFV replication cycle starts with entry into the host cell by dynamin- and clathrin-mediated endocytosis via unknown virus-specific binding receptors or non-specific micropinocytosis [55, 56], followed by internalisation via the endocytic pathway, where sequential viral uncoating occurs. The uncoated ASFV particle reaches the perinuclear area in the cytoplasm and releases viral genome for replication [57]. At the early phase of genome replication, the ASFV DNA briefly transports into and out of the nucleus with the help of ASFV proteins p14 and p37 [9, 58]. Transcription of ASFV genes is initiated before viral genome replication, with both chains of the double-stranded ASFV genome able to alternatively serve as the coding template [59, 60]. The assembly process of ASFV virions occurs in the cytoplasm in viral factories which are held together by a network of microtubules, located near the nucleus and surrounded by ER membranes [61, 62]. Viral proteins and nucleic acids are retained in the viral factories where they interact to form the nucleoid (p10 and pA104R), inner core shell (p35, p15, p8, p150, p37, p34, p14 and p5), inner envelope (p12, p17, pE183L, pE248R, pH108R, pE199L and p22), outer capsid (p72, pE120R and pB438L) and outer envelope (p12 and pE402R) structures that are eventually assembled into a mature virion [63, 64]. At the end of the replication cycle, mature ASFV virions are transported from the viral assembly sites to the plasma membrane by microtubules, then exit the host cell by budding [61]. *This diagram was created with bioRENDER*.

Monocytes and macrophages are the primary cell targets of ASFV [54]. The ASFV replication cycle (Figure 1) starts with entry into the host cell, which can be facilitated by multiple ways, including specific receptor-mediated endocytosis and non-specific micropinocytosis [55, 56], which is followed by virus uncoating and release of viral genomic material for the preparation of replication [57]. During the early phase of genome replication, the viral DNA is transiently present in the nucleus [9, 58]. Expression of ASFV genes is initiated prior to the onset of viral genome replication in so-called viral factories adjacent to the nucleus [59, 60]. Viral structural proteins and nucleic acids are then recruited to the viral factories [61, 62], which are assembled into mature virions [63, 64] and then transported to the plasma membrane, where budding out of the cell occurs [61].

### 4.1. Gene Targets to Produce DISC ASFVs

Possible gene targets for the development of DISC ASF vaccines are listed in Table 2. The proteins encoded by these genes have various functions and are involved in different stages of the replication cycle, including virus internalisation [65], downregulation of the host metabolic pathways [66, 67], genome replication [68, 69], viral gene transcription [70, 71] and morphogenesis [43, 72–76]. The deletion of late genes that are involved in virus assembly has shown promise in producing non-infectious viral particles. For example, suppressed expression of a chaperone protein pB602L aborted the formation of ASF virions. However, the absence of virions coincided with the absence of the major capsid protein p72, which may compromise the immunogenicity of a DISC virus targeting the *B602L* gene [72]. Similarly, several other morphogenesis-related genes have been identified as crucial for virion formation [73–76]. However, potential loss of immunogenic antigens that are important for protective immunity should be taken into consideration when we decide to target viral genes that are necessary for viral assembly.

Other known ASFV genes that have not been studied for their functions in the replication cycle are listed in Table S1. The process of identifying replication-essential viral genes may be limited if only based on the published studies on ASF virology. There are new technologies available to maximise the capability of identifying and shortlisting essential genes in a comprehensive way. Genome-wide gene knock-down is one such tool for this purpose that is independent of prior knowledge. However, preparation of small interfering RNAs (siRNAs) for genome-scale libraries is recognised as costly and labour-intensive, potentially limiting the application of this approach [77].

Regardless of which genes are to be considered for deletion from the ASFV genome to make a DISC virus, it will be necessary to evaluate residual virulence of the DISC virus in vitro and in vivo, as well as the levels of immunogenicity of the viral proteins produced from only one cycle of replication, in order to confirm the DISC virus is a safe and efficacious vaccine candidate.

### 4.2. Novel Genetic Engineering Technologies to Produce DISC ASFVs

The application of CRISPR/Cas9 for knocking out non-essential genes from the ASFV has significantly increased the recombination efficiency compared to traditional homologous recombination techniques [78]. However, although the recombination rate using CRISPR/Cas9 was a significant improvement on conventional homologous recombination, it was still less than 1%. Recent advances in programmable RNA-guided genome editing, such as the IS110 bridge recombination system, which uses a recombinase to drive the expression of a non-coding RNA with binding loops for recognising specific nucleotide sequences, may have potential application to ASFV genome engineering to improve the efficiency of DNA recombination [79].

Bacterial artificial chromosomes (BACs) and yeast artificial chromosomes (YACs) are capable of harbouring large viral genomes and have potential for cloning ASFV genomes that can be utilised for developing DISC viruses [80, 81]. By using the BAC or YAC method, genetic modifications on the viral genome are independent from virus production, thereby minimising the likelihood of unintended changes in the recombinant virus. Drawing from their applications in other large DNA viruses, such as VACV and human cytomegalovirus (HCMV), reconstitution of viable virus from genomic DNA generally requires a helper virus, which allows the virus genome to utilise the virus replication machinery to complete its own cycle and to be packaged into infectious virions [82, 83]. Recently, BAC technology was used to engineer the entire genome of ASFV-Kenya-IX-1033. Viable recombinant virus was rescued by using either homologous or heterologous (genotype 2) ASFV as the helper virus with CRISPR-Cas9 employed to inhibit replication of the helper virus, thereby facilitating the isolation of the rescued mutant virus [80].

### 4.3. DIVA (Differentiating Infected From Vaccinated Animals) Capability for Diagnostics

To enable serological DIVA, incorporation of immunogenic markers into DISC vaccines is an important feature. Vaccines can be formulated to become DIVA compatible by including a unique or foreign immunogenic protein sequence that is effective for DIVA, safe to be used in animals and does not adversely affect subsequent vaccinations [84]. As well as insertion of a foreign immunogenic peptide sequence, the absence of at least one immunogenic viral protein in the vaccine (e.g., by gene deletion or knock-out), allows DIVA via negative detection of antibodies to that antigen [85, 86], as for subunit or inactivated veterinary vaccines, where negative serology for non-structural proteins indicates past immunisation.

## 5. Challenges in the Development of a DISC Vaccine for ASF

### 5.1. Development of a Continuous Cell Line for Productive Infection

Natural target cells for ASFV infection include primary swine monocytes and macrophages of lung, kidney, secondary lymphoid organs and bone marrow-derived macrophages. While there are a range of non-monocyte/macrophage cell lines that are susceptible to ASFV infection, they have varying levels of susceptibility [87]. Primary porcine cells are the gold standard for the isolation and propagation of ASFV; however, their use for vaccine production has drawbacks. The process of preparing primary porcine cells is time-consuming, costly and requires specialised tissue culture facilities. Batch-to-batch variation in sensitivity for ASFV propagation can occur, and unless specific pathogen-free pigs are used, there can be a risk of contamination with adventitious agents or other swine pathogens. The requirements to maintain or have access to animal facilities, along with animal ethics approval and associated compliance further restrict their use and add to the cost of manufacture [87]. A continuous cell line that is permissive to infection by a broad spectrum of ASFV isolates and types and that is cost-effective to culture, and maintain is therefore desirable for ASFV vaccine development.

Continuous porcine monocyte cell lines have been developed and evaluated for their ability to support ASFV replication. A porcine alveolar macrophage (PAM) cell line ZMAC-4, was shown to be susceptible to eight different field isolates of ASFV with comparable levels of replication kinetics and infectious yields to porcine bone marrow cells (PBMCs) [88].Moreover, serial passage of the naturally attenuated isolate OURT88/3 in ZMAC-4 cells did not adversely affect the induction of protective immunity against challenge with the virulent OURT88/1 isolate [88]. However, the growth of ZMAC-4 cells requires media supplementation with macrophage colony-stimulating factor, which may add to costs associated with the potential application of these cells to vaccine production [88]. Other continuous cell lines derived from immortalised PAMs (IPAM), such as 3D4/2, 3D4/21 and 3D4/31 cells, were developed by clonal purification after transfecting primary cells with the SV40 large T antigen gene [89]. These cells are able to support efficient replication of a Vero cell-adapted ASFV strain Lisbon/61, but to a lower degree for isolate Lillie SI/85, compared to Vero cells or PBMCs. Recent studies of the 3D4/21 cell line for its susceptibility to a range of ASFV isolates have shown that this cell line is sensitive to only some isolates and does not support productive infection with genotype 2 strains [90, 91]. Wild boar lung-derived WSL cells are another available continuous porcine cell line that was spontaneously immortalised [54]. WSL cells can support the growth of various ASFVs belonging to genotypes 1, 2 or 9 to comparable levels of PAM [92].Porcine intestinal macrophages (IPIMs) and porcine kidney macrophages (IPKMs) were successfully immortalised by transfecting isolated primary macrophages with the lentiviral-vectored SV40 large T antigen gene and porcine telomerase reverse transcriptase gene [93, 94]. The immortalised IPIM and IPKM cell lines have been shown to be permissive to three different ASFV isolates with comparable or better replication kinetics and infectious yields to primary PAMs [93, 95].

Manipulation of continuous cell lines by transgenic expression of cellular receptors has been an effective approach to generating permissive porcine cells for porcine reproductive and respiratory syndrome virus infection [96]. Identification of cell receptors for ASFV may facilitate a similar approach. By comparing transcriptional profiles of ASFV susceptible and less/non-susceptible cell lines (PK15 and 3D4/21), membrane proteins CD163 and Siglec1 (CD169) were found to be critical for ASFV infection in vitro [97]. However, over-expression of CD163 and Siglec1 in combination was required for productive infection, indicating a synergistic role for these proteins in ASFV cell entry and infection. Evaluation of the expression of macrophage cell markers with levels of ASFV infection in PBMCs also revealed that CD45^+^ and MHCII^+^ cells are strongly associated with infection, while CD163^+^ and CD203a^+^ expression is non-essential [98]. High-throughput genome-wide screening of host cells is a useful tool to identify other cellular factors required for ASFV replication. A genome-wide CRISPR/Cas9 knockout screen in porcine cells indicated the host genes encoding major histocompatibility complex II proteins are important throughout the ASFV replication cycle [99]. Further investigation of virus-host interactions at a cellular level to identify essential host factors to ASFV replication is a key to enabling the development of continuous cell lines that can be employed as suitable and scalable cell substrates to recover and propagate DISC ASF vaccine candidates.

### 5.2. Variations in Characteristics Among Different Strains of ASFV

Selection of any vaccine strain should consider circulating ASFV strains to ensure immunisation will provide adequate levels of protection in pigs. Virological characteristics such as genotype, virulence, haemadsorption phenotype and replication in different cell lines, which can vary significantly among different isolates of ASFV, should also be taken into consideration. Field isolates from clinical specimens typically require cell adaptation to enable genetic manipulation required to engineer a DISC virus. Cell culture-adapted ASFVs have been developed as a workaround for the limited availability of well-characterised and permissive porcine cell lines. Vero cells are a well-established continuous cell line derived from the African green monkey kidney that have been used for adaptation of ASFV strains, including an isolate of the pandemic genotype 2 strain, Georgia/07 and the genotype 1 isolate BA71 (BA71V) [14, 100]. Another African green monkey kidney-derived cell line MA-104, has also been evaluated for isolation and propagation of ASFV from field specimens [101]. However, cell adaptation typically leads to major deletions in the ASFV genome that result in significant attenuation of the virus [14, 102], which may limit the application of cell-adapted viruses for DISC virus vaccine development.

While cell-adapted viruses can be a convenient tool for the study of ASF virology, they may not be representative of circulating wild type strains because of phenotypic and genomic differences. Nevertheless, adaptation of existing LAV vaccine strains to continuous cell lines is a promising approach to achieve the goal of a vaccine cell substrate suitable for scalable vaccine manufacture [103]. As mentioned before, PIPEC has been used for adaptation of ASFV-G-ΔI177L to generate ASFV-G-ΔI177L/ΔLVR with a deletion in the LVR as another LAV vaccine [38]. Such an approach may also be applied to the development of DISC ASFVs, provided adaptation does not lead to major alterations to the viral genome that affect replicative and antigenic characteristics.

Discrepancies in reported replication essential genes for different ASFV strains also complicate target selection for the development of a DISC ASFV. For example, a histone-like protein encoded by *A104R* that is associated with DNA binding/packaging has been shown to be essential to the replication of BA71V [42, 43], while it is not essential for ASFV Georgia 2010, but its deletion reduces virulence in pigs infected with the latter [104]. This finding highlights the uncertain translatability of a surrogate strain to circulating or wild type ASFV isolates and the importance of confirming the function of viral gene targets during DISC vaccine development.

### 5.3. Reversion to Virulence or Replication-Competency

Reversion to virulence is a safety concern for attenuated ASFVs due to the evolutionary nature of the ASFV genome [6, 105]. Notably, mutations in the C257L protein of one of the licenced LAV vaccines in Vietnam, ASFV-G-ΔI177L, were confirmed after serial passage in pigs for up to four times, resulting in reversion to virulence that caused severe ASF-related clinical signs in pregnant sows [39]. Discovery of a highly lethal genotype 1 and 2 recombinant strain of ASFV in China and Vietnam also highlights the risk of recombination between a co-circulating attenuated virus (genotype 1) with a virulent virus (genotype 2) to form a new virulent recombinant strain that is lethal for pigs immunised with a genotype 2 LAV [6, 106]. Similarly, DISC viruses could also be subject to the risk of reversion to virulence due to recombination in the scenario where a DISC-vaccine vaccinated pig is co-infected with a wild type ASFV before the vaccine has been cleared from the body.

## 6. Conclusion

Development of a vaccine solution for ASF that fulfils desired safety and immunogenicity characteristics is inherently challenging. While first-generation ASF LAVs offer a major advancement, and for the first time, provide a valuable tool to prevent ASF, safety concerns with replication-competent vaccines warrant the development of safer alternatives that can be utilised for a wider range of applications. Among these alternative approaches, replication-defective DISC viruses have the potential to provide the spectrum of authentic ASFV proteins required for robust host immunity. Identification of essential viral gene(s) as deletion targets that maintain the immunogenicity of the selected vaccine strain is key to the successful development of a DISC ASFV. Selected gene(s) should be validated for their replicative role (ideally, in vitro and in vivo), while characterisation of the many ASFV genes that have no known function is expected to lead to the identification of additional deletion targets. Technical challenges due to the complexity of ASFVs and limited availability of porcine continuous cell lines can further complicate the development process. Future investigations directed towards the confirmation of possible viral genes as deletion targets and building on existing knowledge of ASFV replication and host response, are expected to provide further guidance for the design of ASFV DISC candidates for a safe next-generation ASF vaccine option.

## Figures and Tables

**Table 1:** Protective live attenuated viruses (LAVs) developed by targeted gene deletion.

Vaccine	Strain	Genotype	Gene(s) deletion	Role of the viral gene(s)	Protection against virus challenge	In vivo safety
ASFV-G-ΔI177L [28]	Georgia 2007/1	2	*I177L*	Function unknown	All 20 vaccinated pigs (10 at 10^2^ HAD_50_^a^, 5 with 10^4^ HAD_50_ and 5 with 10^6^ HAD_50_) were protected against lethal challenge with 10^2^ HAD_50_ parental virus	Low levels of viremia detected until day 28 post, but no virus shedding or clinical signs

ASFV-G-ΔA137R [29]	Georgia 2007/1	2	*A137R*	Encodes a structural protein	All 5 pigs vaccinated with 10^2^ HAD_50_ survived lethal challenge with 10^2^ HAD_50_ parental virus	Low-to-moderate levels of viremia detected until day 21 with no clinical signs. Transmission to cohabitated naïve pigs

ASFV-G-ΔMGF [30]	Georgia 2007/1	2	Six genes belonging to multigene family (MGF) 360 or 505	Host range specificity, blocking host innate immunity, virulence related	All vaccinated pigs (10 with 10^2^ HAD_50_ and 10 with 10^4^ HAD_50_) were protected against lethal challenge with 10^3^ HAD_50_ parental virus	Low-to-high levels of viremia were detected until day 28 post with no clinical signs

BA71ΔCD2 [31]	BA71	1	*EP402R*	Haemadsorption, virulence related	Dose-dependent protection of 2/6 pigs vaccinated with 10^3^ PFU^b^, all 6 pigs with 3.3x 10^4^ PFU and all 6 pigs with 10^6^ PFU, against lethal challenge with 10^3^ HAD_50_ parental virus	No detectable viremia and no clinical signs
					Dose-dependent protection of 1/6 pigs vaccinated with 10^3^ PFU, all 6 pigs with 3.3x 10^4^ PFU and all 6 with 10^6^ PFU, against lethal challenge with 10^4^ HAD_50_ heterologous strain E75	

ASFV-G-Δ9GL/ΔUK [32]	Georgia 2007/1	2	*B119L* and *DP96R*	Functions unknown	Four out of nine pigs vaccinated with 10^2^ HAD_50_, all 10 pigs vaccinated with 10^4^ HAD_50_, and 14/15 with 10^6^ HAD_50_, survived lethal challenge with 10^3^ HAD_50_ parental virus	Low-to-high levels of viremia peaked by around day 7, decreased to moderate levels by day 21, no clinical signs

BeninΔDP148R [33]	Benin 97/1	1	*DP148R*	Function unknown	All 5 pigs vaccinated with 10^3^ HAD_50_ survived lethal challenge with 10^4^ HAD_50_ parental virus	Moderate levels of viremia detected until day 35 with transient clinical symptoms

HLJ/18–7GD [34]	HLJ/2018	2	Six genes belonging to MFG505 or 360, and *EP402R*	Haemadsorption, host range specificity, blocking of host innate immunity, virulence related	All vaccinated pigs (4 with 10^3^ TCID_50_^c^ and 4 with 10^5^ TCID_50_) survived lethal challenge with 200 PLD_50_^d^ parental virus	Moderate levels of viremia with transient clinical signs

^a^HAD_50_: median haemadsorption units.

^b^PFU: plaque forming unit.

^c^TCID_50_: 50% tissue culture infectious dose.

^d^PLD_50_: 50% pig lethal dose.

**Table 2:** Possible gene targets for deletion to produce ASF DISC vaccines.

Gene(s)	Protein(s) encoded	Replication function	Essential function confirmed by
*CP204L* [65]	p30 (p32) phosphoprotein	Host interaction: interacts with host protein VPS39, mediating virus internalisation	CRISPR/Cas9 targeting *CP204L* showed efficient inhibition of virus replication

*Ep152R* [66]	Ep152R early protein	Host interaction: interacts with host-protein BAG6 to facilitate viral replication	Recombinant ASFV with deletion of *EP152R* could not be recovered using either Georgia or BA71V as the parental strain, suggesting an essential function of the viral gene

*I215L* [67]	pI215L ubiquitin-conjugating enzyme	Host interaction: interacts with host protein RING finger protein 138 to inhibit type I interferon production	Knockdown of *I215L* expression inhibited ASFV replication

*E301R* [68]	pE301R proliferating cell nuclear antigen-like protein	Genome replication: functions as a sliding clamp to secure DNA polymerase to the DNA molecule to initiate viral DNA replication	Small interfering RNA (siRNA) knockdown significantly reduced viral genome copy numbers, indicating its essential role to replication

*E296R* [69]	pE296R apurinic/apyrimidinic endonuclease	Genome replication: repairs mismatches in viral DNA	A *E296R* deletion mutant has been constructed from BA71V. While the growth kinetics of the mutant showed no difference to the parental virus in Vero cells, it was reduced significantly in swine macrophages, suggesting an essential function for ASFV infection in the natural host

*D1133L* [70]	Helicase superfamily II	Gene transcription: involved in transcription initiation; interacts with host proteins for cellular activities to facilitate viral replication	A high affinity drug compound for this viral protein downregulated its expression and inhibited ASFV replication in vitro

*Q706L* and *QP509L* [71]	Encode RNA helicases	Gene transcription: involved in transcription initiation	siRNA knockdown of either gene significantly reduced production of infectious progeny

*B602L* [72]	pB602L	Morphogenesis: chaperone protein for p72	Repression of *B602L* led to the production of abnormal forms of virions

*B438L* [73]	pB438L structural protein	Morphogenesis: forms a part of the virus membrane	Inhibition of *B438L* led to the production of abnormal forms of viral proteins

*EP84R* [74]	pEP84R structural protein	Morphogenesis: forms a part of the virus membrane	Inhibition of *EP84R* led to the production of viral particles that have no nucleoid, which are still able to exit the cell

*E183L* [75]	p54 structural protein	Morphogenesis: affects microtubular organisation during infection, associated with transportation of virus and viral proteins within the cell	Abnormal structures of virions (zipper-like structures) were observed with inhibited expression of *E183L*

*E248R* [76]	pE248R structural protein	Morphogenesis: a component of the virus membrane	Viral particles produced after repression of this membrane protein are indistinguishable from the wild-type virions and can exit the cell normally, however, infectivity of pE248R-deficient virions was significantly reduced

*A104R* [43]	pA104R histone-like protein	Morphogenesis: involved in viral genome packaging	siRNA knockdown significantly reduced viral progeny numbers, indicating an essential role in replication

## Data Availability

The data sharing is not applicable to this article as no new data were created or analysed in this study.

## Supporting Information

Supporting Information Table S1. Other known ASFV genes to be further investigated for essential functional roles.
