# Supplementary material for: The Potential of Disabled Infectious Single Cycle (DISC) Virus Platforms for Next Generation African Swine Fever Vaccine Development
Source: Transbound Emerg Dis. 2025 Jul 14;2025:8573171. doi: 10.1155/tbed/8573171 (PMC12279434; doi:10.1155/tbed/8573171)
Supplement: Supporting Information — Table S1. Other known ASFV genes to be further investigated for essential functional roles. [file 8573171.f1.docx]

**Supplementary Table 1. Other known ASFV genes to be further investigated for essential functional roles.**

| **Gene(s)** | **Protein(s) encoded** | **Function involved in replication** |
| --- | --- | --- |
| *G1211R* [1] | pG1211R B family DNA polymerase | Genome replication: initiates DNA replication |
| *F1055L* [2, 3] | Encodes primase and helicase domains | Genome replication: putative involvement in initiating DNA replication |
| *F334L* and *F778R* [4] | Respectively encode the small and the large subunits of a ribonucleotide reductase | Genome replication: involved in viral DNA and nucleotides synthesis |
| *NP419L* [5] | DNA ligase | Genome replication: putative involvement in DNA repair following recombination |
| *EP364R* [5, 6] | pEP264R nuclease | Genome replication: putative involvement in DNA repair following recombination  Host interaction: inhibits type I interferon production |
| *D345L* [7] | pD345L exonuclease | Genome replication: putative involvement in DNA repair following recombination  Host interaction: inhibits type I interferon production |
| *CP2475L* [8-10] | Polyprotein pp220 | Morphogenesis: pp220 is processed into p150, p37, p14, p5 and p34, which are part of the viral inner core shell structure   Genome replication: p37 and p14 are involved in nuclear transport of viral DNA during early stage of replication |
| *K78R* [11] | p10 structural protein | Immunogenicity: induces antibody response  Genome replication: involved in nuclear transport of viral DNA during early stage of replication |
| *C315R* [12] | Encodes a transcription factor | Gene transcription: involved in transcription initiation and RNA polymerase recruitment |
| *EP1242L, D339L, C147L, NP1450L, H359L, D205R, CP80R* and *C105R* [12, 13] | Encode subunits (RPB2, RPB7, RPB6, RPB1, RPB3-11 fusion, RPB5, RPB10, and RPB9, respectively) of an RNA polymerase | Gene transcription |
| *M448R* [14] | Putative RNA ligase | Gene transcription: putative involvement in RNA repair activity  Immunogenicity: recognised by ASFV-specific T cells |
| *I243L* [15] | Encodes a transcription factor | Gene transcription: involved in transcription initiation |
| *B962L* [16] | Putative RNA helicase | Gene transcription: involved in transcription initiation |
| *NP868R* [17] | Encodes an enzyme for capping viral mRNAs | Gene transcription: involved in transcription and pre-translation |
| *C475L* [5] | Putative polyA polymerase | Gene transcription: involved in polyadenylation for mRNA maturation |
| *B263R* [18] | Putative transcription factor | Gene transcription: involved in transcription initiation |
| *E423R* [19] | Transcription factor | Gene transcription |
| *B385R* [5] | Putative transcription factor | Gene transcription |
| *EP424R* [5] | Putative RNA methyltransferase | Viral protein expression: helps stabilise ribosomal RNA for viral protein synthesis |
| *CP530R* [10, 20] | Polyprotein pp62 | Morphogenesis: pp62 is processed into p35, p15 and p8, which are part of the viral inner core shell structure; p15 has DNA binding properties for unknown purpose |
| *B354L* [21, 22] | pB354L ATPase | Morphogenesis: putative involvement in packaging viral DNA into virions |
| *P1192R* [23, 24] | Putative DNA topoisomerase | Morphogenesis: facilitates viral genome segregation during viral assembly |
| *D250R* [25] | Nudix hydrolase | Morphogenesis: involved in regulating viral morphogenesis, especially viral factory organisation |
| *CP123L* [26] | pCP123L membrane protein | Immunogenicity: induces both antibody and T cell responses |
| *XP124L* [27] | pXP124L, belongs to Multigene Family 110 proteins | Morphogenesis: putative involvement in the formation of viral factories |
| *H171R* and *C257L* [28] | Small structural proteins | Morphogenesis: may be associated with stabilisation of virions |
| *B117L* [29] | pB117L small structural protein | Host interaction: putative involvement in virus entry |
| *D117L* [30, 31] | p17 structural protein | Host interaction: inhibits type I interferon signalling pathways |
| *E199L [32]* | j18L structural protein | Morphogenesis: part of the viral membrane  Host interaction: mediates virus entry |
| *E120R* [33, 34] | p14.5 structural protein | Morphogenesis: part of the capsid structure, involved in transportation of assembled virus to plasma membrane  Host interaction: inhibits type I interferon production |
| *CP312R* [35, 36] | pCR312R structural protein | Immunogenicity: induces antibody responses  Host interaction: interacts with host protein RPS27A to inhibit host protein synthesis to promote viral replication |
| *B646L* [5] | p72 major structural protein | Morphogenesis: part of the capsid structure  Host interaction: associated with virus attachment to the host cell |
| *O61R* [37, 38] | p12 structural protein | Immunogenicity: induces antibody responses  Host interaction: associated with attachment for virus entry |
| *S273R* [39] | pS273R protease | Host interaction: inhibits type I interferon production  Morphogenesis: cleaves ASFV pp62 and pp220 |
| *G1340L* [40, 41] | Possibly encodes a transcription factor | Host interaction: inhibits type I interferon production  Immunogenicity: induces cytotoxic T cell responses |
| *B175L* [42] | Possibly encodes a transcription factor | Host interaction: inhibits type I interferon mediated signalling responses |
| *B318L* [43] | Trans-geranylgeranyl-diphosphate synthase | Host interaction: inhibits type I interferon mediated signalling responses |
| *R298L* [4] | Serine/threonine protein kinase | Host interaction: involved in the regulation of host cell cycle to help virus gain the nucleotides synthesised by the cell |
| *EP364R* [6] | Encodes a nuclease | Host interaction: inhibits type I interferon signalling pathways |
| *K421R* [44] | Not identified | Host interaction: inhibits type I interferon signalling pathways |
| *B475L* [45] | pB475L | Host interaction: inhibits type I interferon signalling pathways |

**References**

[1] L. Kuai, J. Sun, Q. Peng, X. Zhao, B. Yuan, S. Liu, Y. Bi, Y. Shi, Cryo-EM structure of DNA polymerase of African swine fever virus, Nucleic Acids Research (2024).

[2] Z. Shao, S. Su, J. Yang, W. Zhang, Y. Gao, X. Zhao, Y. Zhang, Q. Shao, C. Cao, H. Li, H. Liu, J. Zhang, J. Lin, J. Ma, J. Gan, Structures and implications of the C962R protein of African swine fever virus, Nucleic Acids Res 51(17) (2023) 9475-9490.

[3] E. Ramirez-Medina, E.A. Vuono, A. Rai, S. Pruitt, E. Silva, L. Velazquez-Salinas, J. Zhu, M.V. Borca, D.P. Gladue, The C962R ORF of African Swine Fever Strain Georgia Is Non-Essential and Not Required for Virulence in Swine, Viruses 12(6) (2020).

[4] H.R. Avagyan, S.A. Hakobyan, A.A. Poghosyan, N.V. Bayramyan, H.H. Arzumanyan, L.O. Abroyan, A.S. Avetisyan, L.A. Hakobyan, E.M. Karalova, Z.A. Karalyan, African Swine Fever Virus Manipulates the Cell Cycle of G0-Infected Cells to Access Cellular Nucleotides, Viruses 14(8) (2022).

[5] L.K. Dixon, D.A.G. Chapman, C.L. Netherton, C. Upton, African swine fever virus replication and genomics, Virus Research 173(1) (2013) 3-14.

[6] N. Dodantenna, L. Ranathunga, W.A.G. Chathuranga, A. Weerawardhana, J.-W. Cha, A. Subasinghe, N. Gamage, D.K. Haluwana, Y. Kim, W. Jheong, H. Poo, J.-S. Lee, African Swine Fever Virus EP364R and C129R Target Cyclic GMP-AMP To Inhibit the cGAS-STING Signaling Pathway, J Virol 96(15) (2022) e01022-22.

[7] H. Chen, Z. Wang, X. Gao, J. Lv, Y. Hu, Y.-S. Jung, S. Zhu, X. Wu, Y. Qian, J. Dai, ASFV pD345L protein negatively regulates NF-κB signalling by inhibiting IKK kinase activity, Veterinary Research 53(1) (2022) 32.

[8] G. Andrés, R. García-Escudero, M.L. Salas, J.M. Rodríguez, Repression of African swine fever virus polyprotein pp220-encoding gene leads to the assembly of icosahedral core-less particles, J Virol 76(6) (2002) 2654-66.

[9] A. Eulálio, I. Nunes-Correia, A.L. Carvalho, C. Faro, V. Citovsky, S. Simões, M.C. Pedroso de Lima, Two African swine fever virus proteins derived from a common precursor exhibit different nucleocytoplasmic transport activities, J Virol 78(18) (2004) 9731-9.

[10] A. Alejo, T. Matamoros, M. Guerra, G. Andrés, A Proteomic Atlas of the African Swine Fever Virus Particle, J Virol 92(23) (2018) 10.1128/jvi.01293-18.

[11] Q. Xie, Y. Bai, W. Wang, R. Chen, H. Xing, Y. Wu, G. Shao, Z. Bu, D. Zhao, Z. Feng, Dynamics of Serological and Mucosal Antibody Responses against African Swine Fever Viruses in Experimentally Infected Pigs, Transboundary and Emerging Diseases 2023(1) (2023) 9959847.

[12] Y. Zhang, Z. Zhang, F. Zhang, J. Zhang, J. Jiao, M. Hou, N. Qian, D. Zhao, X. Zheng, X. Tan, ASFV transcription reporter screening system identifies ailanthone as a broad antiviral compound, Virologica Sinica 38(3) (2023) 459-469.

[13] G. Cackett, M. Sýkora, F. Werner, Transcriptome view of a killer: African swine fever virus, Biochemical Society Transactions 48(4) (2020) 1569-1581.

[14] L. Bosch-Camós, E. López, J. Collado, M.J. Navas, M. Blanco-Fuertes, S. Pina-Pedrero, F. Accensi, M.L. Salas, E. Mundt, V. Nikolin, F. Rodríguez, M448R and MGF505-7R: Two African Swine Fever Virus Antigens Commonly Recognized by ASFV-Specific T-Cells and with Protective Potential, Vaccines 9(5) (2021) 508.

[15] J.M. Rodríguez, M.L. Salas, E. Viñuela, Intermediate class of mRNAs in African swine fever virus, J Virol 70(12) (1996) 8584-8589.

[16] R.J. Yáñez, J.M. Rodríguez, M. Boursnell, J. Rodriguez, E. Viñuela, Two putative African swine fever virus helicases similar to yeast ‘DEAH’ pre-mRNA processing proteins and vaccinia virus ATPases D11L and D6R, Gene 134(2) (1993) 161-174.

[17] H.E. Eaton, T. Kobayashi, T.S. Dermody, R.N. Johnston, P.H. Jais, M. Shmulevitz, African Swine Fever Virus NP868R Capping Enzyme Promotes Reovirus Rescue during Reverse Genetics by Promoting Reovirus Protein Expression, Virion Assembly, and RNA Incorporation into Infectious Virions, J Virol 91(11) (2017).

[18] D. Kinyanyi, G. Obiero, G.F.O. Obiero, P. Amwayi, S. Mwaniki, M. Wamalwa, In silico structural and functional prediction of African swine fever virus protein-B263R reveals features of a TATA-binding protein, PeerJ 6 (2018) e4396.

[19] J.M. Rodríguez, L.T. Moreno, A. Alejo, A. Lacasta, F. Rodríguez, M.L. Salas, Genome Sequence of African Swine Fever Virus BA71, the Virulent Parental Strain of the Nonpathogenic and Tissue-Culture Adapted BA71V, PLOS ONE 10(11) (2015) e0142889.

[20] F. Guo, Y. Shi, M. Yang, Y. Guo, Z. Shen, M. Li, Y. Chen, R. Liang, Y. Yang, H. Chen, G. Peng, The structural basis of African swine fever virus core shell protein p15 binding to DNA, The FASEB Journal 35(3) (2021) e21350.

[21] M. Iyer Lakshminarayan, L. Aravind, V. Koonin Eugene, Common Origin of Four Diverse Families of Large Eukaryotic DNA Viruses, J Virol 75(23) (2001) 11720-11734.

[22] F.-Y. Lin, K.-W. Chan, H.-C. Wang, W.-L. Hsu, M.-L. Wong, Functional expression of the recombinant ATPase of orf virus, Archives of Virology 155(10) (2010) 1701-1705.

[23] S.A. Baylis, L.K. Dixon, S. Vydelingum, G.L. Smith, African swine fever virus encodes a gene with extensive homology to type II DNA topoisomerases, J Mol Biol 228(3) (1992) 1003-10.

[24] J. Coelho, C. Martins, F. Ferreira, A. Leitão, African swine fever virus ORF P1192R codes for a functional type II DNA topoisomerase, Virology 474 (2015) 82-93.

[25] J.L. Cartwright, S.T. Safrany, L.K. Dixon, E. Darzynkiewicz, J. Stepinski, R. Burke, A.G. McLennan, The g5R (D250) Gene of African Swine Fever Virus Encodes a Nudix Hydrolase That Preferentially Degrades Diphosphoinositol Polyphosphates, J Virol 76(3) (2002) 1415-1421.

[26] L.C. Goatley, P. Tng, L. Al-Adwani, Z. Hargreaves, S. Levin, T. Lambe, C.L. Netherton, ASFV antigens selected from genotype I immunised pigs are immunogenic, but do not protect against genotype II challenge, Veterinary Vaccine 2(3) (2023) 100042.

[27] C. Netherton, I. Rouiller, T. Wileman, The subcellular distribution of multigene family 110 proteins of African swine fever virus is determined by differences in C-terminal KDEL endoplasmic reticulum retention motifs, J Virol 78(7) (2004) 3710-21.

[28] Z. Shen, C. Chen, Y. Yang, Z. Xie, Q. Ao, L. Lv, S. Zhang, H. Chen, R. Hu, H. Chen, G. Peng, Novel Function of African Swine Fever Virus pE66L in Inhibition of Host Translation by the PKR/eIF2α Pathway, J Virol 95(5) (2021) 10.1128/jvi.01872-20.

[29] D.P. Gladue, L. Gomez-Lucas, E. Largo, L. Velazquez-Salinas, E. Ramirez-Medina, J. Torralba, M. Queralt, A. Alcaraz, J.L. Nieva, M.V. Borca, African Swine Fever Virus Gene B117L Encodes a Small Protein Endowed with Low-pH-Dependent Membrane Permeabilizing Activity, J Virol 97(6) (2023) e00350-23.

[30] C. Suárez, J. Gutiérrez-Berzal, G. Andrés, M.L. Salas, J.M. Rodríguez, African swine fever virus protein p17 is essential for the progression of viral membrane precursors toward icosahedral intermediates, J Virol 84(15) (2010) 7484-99.

[31] W. Zheng, N. Xia, J. Zhang, Q. Cao, S. Jiang, J. Luo, H. Wang, N. Chen, Q. Zhang, F. Meurens, J. Zhu, African Swine Fever Virus Structural Protein p17 Inhibits cGAS-STING Signaling Pathway Through Interacting With STING, Frontiers in Immunology 13 (2022).

[32] T. Matamoros, A. Alejo, J.M. Rodríguez, B. Hernáez, M. Guerra, A. Fraile-Ramos, G. Andrés, African Swine Fever Virus Protein pE199L Mediates Virus Entry by Enabling Membrane Fusion and Core Penetration, mBio 11(4) (2020).

[33] G. Andrés, R. García-Escudero, E. Viñuela, M.L. Salas, J.M. Rodríguez, African swine fever virus structural protein pE120R is essential for virus transport from assembly sites to plasma membrane but not for infectivity, J Virol 75(15) (2001) 6758-68.

[34] S. Cui, Y. Wang, S. Chen, L. Fang, Y. Jiang, Z. Pang, Y. Jiang, X. Guo, H. Zhu, H. Jia, African swine fever virus E120R inhibited cGAS-STING-mediated IFN-β and NF-κB pathways, Animal Research and One Health 2(1) (2024) 39-49.

[35] Y.T. Hagoss, D. Shen, W. Wang, Z. Zhang, F. Li, E. Sun, Y. Zhu, J. Ge, Y. Guo, Z. Bu, D. Zhao, African swine fever virus pCP312R interacts with host RPS27A to shut off host protein translation and promotes viral replication, International Journal of Biological Macromolecules 277 (2024) 134213.

[36] Y.T. Hagoss, D. Shen, Z. Zhang, F. Li, Z. Bu, D. Zhao, Novel Epitopes Mapping of African Swine Fever Virus CP312R Protein Using Monoclonal Antibodies, Viruses 15(2) (2023).

[37] M.L. Salas, G. Andrés, African swine fever virus morphogenesis, Virus Research 173(1) (2013) 29-41.

[38] Z. Xu, Y. Hu, J. Li, A. Wang, X. Meng, L. Chen, J. Wei, W. Tong, N. Kong, L. Yu, H. Yu, T. Shan, G. Tong, G. Wang, H. Zheng, Screening and identification of the dominant antigens of the African swine fever virus, Frontiers in Veterinary Science 10 (2023).

[39] H. Li, X. Zheng, Y. Li, Y. Zhu, Y. Xu, Z. Yu, W.H. Feng, African swine fever virus S273R protein antagonizes type I interferon production by interfering with TBK1 and IRF3 interaction, Virol Sin 38(6) (2023) 911-921.

[40] A. Leitão, A. Malur, C. Cartaxeiro, G. Vasco, B. Cruz, P. Cornelis, C.L.V. Martins, Bacterial lipoprotein based expression vectors as tools for the characterisation of African swine fever virus (ASFV) antigens, Archives of Virology 145(8) (2000) 1639-1657.

[41] Q. Wu, Y. Lei, Y. Zuo, J. Zhang, F. Guo, W. Xu, T. Xie, D. Wang, G. Peng, X. Wang, H. Chen, Z. Fu, G. Cao, J. Dai, Interactome between ASFV and host immune pathway proteins, mSystems 8(6) (2023) e00471-23.

[42] L. Ranathunga, N. Dodantenna, J.-W. Cha, K. Chathuranga, W.A.G. Chathuranga, A. Weerawardhana, A. Subasinghe, D.K. Haluwana, N. Gamage, J.-S. Lee, African swine fever virus B175L inhibits the type I interferon pathway by targeting STING and 2′3′-cGAMP, J Virol 97(11) (2023) e00795-23.

[43] X. Liu, H. Chen, G. Ye, H. Liu, C. Feng, W. Chen, L. Hu, Q. Zhou, Z. Zhang, J. Li, X. Zhang, X. He, Y. Guan, Z. Wu, D. Zhao, Z. Bu, C. Weng, L. Huang, African swine fever virus pB318L, a trans-geranylgeranyl-diphosphate synthase, negatively regulates cGAS-STING and IFNAR-JAK-STAT signaling pathways, PLOS Pathogens 20(4) (2024) e1012136.

[44] Q. Wu, Y. Lei, Y. Zuo, J. Zhang, F. Guo, W. Xu, T. Xie, D. Wang, G. Peng, X. Wang, H. Chen, Z. Fu, G. Cao, J. Dai, Interactome between ASFV and host immune pathway proteins, mSystems 8(6) (2023) e0047123.

[45] Z. Huang, Z. Mai, C. Kong, J. You, S. Lin, C. Gao, W. Zhang, X. Chen, Q. Xie, H. Wang, S. Tang, P. Zhou, L. Gong, G. Zhang, African swine fever virus pB475L evades host antiviral innate immunity via targeting STAT2 to inhibit IFN-I signaling, Journal of Biological Chemistry 300(7) (2024).
